# Supplementary material for: Waves and currents decrease the available space in a salmon cage
Source: PLoS One. 2022 Feb 25;17(2):e0263850. doi: 10.1371/journal.pone.0263850 (PMC8880890; doi:10.1371/journal.pone.0263850)
Supplement: Attachment — Submitted filename: Rebuttal letter.pdf [file pone.0263850.s001.pdf]

# 1 Rebuttal letter

The authors thank the reviewers for insightful comments, which have improved the manuscript substantially. Please see comments below with our responses in italics and quotes from the changed manuscript are indented.

## 1.1 Reviewer 1

**L28:** A previous work (Johansson et al. 2014) investigated the swimming behaviour of Atlantic salmon at a commercial farm with tidal currents. Please add this reference and related information in the text. I also suggest that this paper must be discussed below and compared to the obtained results in detail.

and

**L31:** I missed some referenced information here about cage deformations by currents and waves, as well as some information about the "regular" behaviour of salmon in cages. Both have been widely studied.

*The authors recognise that the omission of this paper was a mistake and do now discuss the effect of currents on salmon behaviour. We have also added some information about cage deformation and "normal" behaviour as it pertains to particularly daylight and currents. As the reviewer rightly pointed out, this has been widely studied*

> The behaviour of salmon in relation to currents and time of day has also been extensively studied. Salmon change their swimming behaviour according to the availability of light in their environment. When lights are deployed at night during winter, salmon will maintain daytime swimming behaviours and navigate to the depth of the lights [1], while they disperse more and decrease swimming activity if no lights are deployed [2]. This behaviours is modified by other environmental factors, such as water temperature [3]. Current speed affects swimming behaviour in two major ways. Strong current decreases aggressive interactions and increases shoal cohesion in raceways [4] and in salmon cages a strong current changes swimming mode from circular to standing on current, that is maintaining position in the cage while swimming against the current [5].
>
> Currents do not only affect the swimming behaviour and energy expenditure of salmon, they also affect the shape of salmon cages. Cage deformation due to current can cause a decrease in cage

> volume of 20% at 0.5 m $s^{-1}$ [6, 7]. While not as thoroughly studied, there are indications that waves too can change the shape of a salmon cage [8].
>
> There is some recent work on how behaviour is affected by waves, mostly investigating vertical preference and swimming effort [9, 10]. However, while there is data on how changes in the available space within a cage affect biomass and salmon welfare [11], the effects of waves on available space and salmon behaviour have not been thoroughly investigated. Now lines 27-46.

**L34-35:** Why ASC and not other certification scheme? Then, the reference is about RSPCA. I suggest to delete "such as ASC".

*While it is true that our reference was to the RSPCA, we believe both standards are worthy of mention as they both affect how farmers manage their farms. We have added the RSPCA's standard in text and now reference both standards*

> This means that there is growing consumer pressure for not only environmental certifications such as ASC (Aquaculture Stewardship Council) [12], but also for assurances that the farms can deliver a minimum welfare standard, such as the RSPCA assured scheme [13]. Now lines 48-51.

**L60:** Only one cage was monitored? Why don't you monitored more cages? I understand that can be a limitation in terms of logistics, but monitoring only one cage you lose repeatability of results and, most important, certainty when drawing conclusions. Could it be possible that other environmental parameters or husbandry procedures were influencing the behaviour of monitored caged fish?

*We recognise that monitoring only one cage limits the conclusions of our study to that one cage. The equipment used for this work was a limited resource and we did not have the opportunity, equipment, or time to equip many cages so well. We prioritised obtaining many data types in higher detail rather than equipping several cages - or even farms - with limited equipment. While unfortunate, the circumstances of carrying out projects in working commercial fish farms using this kind of equipment is limiting. We have mentioned this limitation in the methods and discuss it further in the discussion.*

> Ideally, several cages would be included from different sites in order to get a general picture of the relationship between waves, currents, and salmon. However, for reasons of logistics, this was not possible with such an extensive setup. Therefore, this study details the conditions in one cage that has been thoroughly monitored. Lines 82-86, methods

> This study is limited by the available study locations. Particularly that only one cage was monitored for salmon behaviour. This limits our conclusion to this one cage, but considering that the current literature on salmon in commercial cages is also often limited to one or a few units, these data add to what is already a limited pool of information. Lines 452-456, discussion

**L67:** what about welfare of lumpfish exposed to currents and waves?

*We consider the welfare of lumpfish to be a very important topic and are involved in projects aimed at improving their welfare. However, studying two species in detail was outside of the scope of this study, which is already resource limited.*

**L119:** Were these OWIs validated before? I miss some references about. These OWIs were checked in salmon and lumpfish? I guess only on salmon. Please clarify.

*These OWIs are based in literature, but adjusted for use in a commercial salmon farm by author Signar P. Dam to make it practically possible to gather the data. We now mention the inspiration for the welfare parameters in the methods:*

> In order to ascertain the general welfare of salmon on the farm during the study period, we carried out Operational Welfare Indicator registrations. During the sampling period, Operational Welfare Indicators (OWIs) were recorded every two weeks when weather allowed (Table **??**). Prior to this, welfare indicators were collected on a more ad-hoc basis to establish a baseline, and finally again at harvest. The large gap in data from March until harvest is due to the COVID-19 pandemic preventing fieldwork. The OWIs were adapted by Hiddenfjord from SWIM 1 and 2 [14,15] to make them more practical to use as part of regular farm management practices. The OWIs were collected from 10 salmon from each cage in connection with routine louse counting. The salmon were caught using a dip net, anaesthetised in 60 mg L $^{-1}$ Finquel (MS-222) and lice numbers and gill condition were recorded before OWIs were recorded. After regaining consciousness in fresh seawater, the salmon were released back into the cages. For the purposes of this study, a sum of scores for each salmon excluding sclera colour was used to determine the overall welfare of the salmon. Sclera colour or eye darkening was not included here because while there is evidence that they can be used as an indicator of stress [16], this has not yet been well established in salmon. The total score excluding sclera can range from 0 to 10 with a low number

indicating good welfare and a high number indicating poor welfare. Many individual based indicators in SWIM 1 and 2 are not included because there was either no variation in them (e.g. sexual maturation did not occur) or they did not relate to conditions on the farm at the time (e.g. deformities). Lines 165-184.

**L123:** Are 10 individuals representative of a fish population of 112 thousand salmon? I don't really think so. What the authors think about this? Are monitored OWIs valid?

*The number of fish collected was limited to the fish that were caught for government mandated louse monitoring. While a sample of 50 fish (ten from each cage) is not a very large number compared to the actual number of animals in the farm, it is what was available to us. These fish were being caught and anaesthetised anyway, so we could monitor them with minimal disruption to work on the farm. In terms of validity, the variation in scores was not very high at this farm, and while a larger sample size might provide robust statistics, but these observations are not made to make big claims about how waves affect welfare.*

**L261:** Discussion section needs some improvements. Although the authors obtained really nice results in this study, but they only compared them with their previous work, as if there were no other work with which to compare and discuss the results obtained. I highly recommend to add in this section every previous works that have been carried out in salmon cages. For example, I miss more information and comparisons with the expected "normal" behaviour of salmon in cages, as well as the effects of currents and waves on the net-pens deformation. There are quite few papers about that match with these results and deserve attention. All these must be discussed and linked with the obtained results in this study.

*The authors acknowledge this rather severe omission of wider literature. We have changed the discussion to put our results into the proper context.*

What about lumpfish behaviour and welfare? Given that the monitored salmon cage held 10 thousand lumpfish, and that the authors address the welfare topic, I believe some lines about how currents and waves can affect this species must be included in the discussion.

*Again, the authors care a great deal about lumpfish welfare, but we chose to not include any data or discussion about them in this manuscript as we feel they deserve studies focused on them alone. However, we do now mention how these conditions may affect lumpfish and underline the necessity to study their welfare in exposed farms further.*

> While currents were weak by 50 cm long salmon standards, these cages were also inhabited by lumpfish, and it is possible that the strongest currents at this site exceed lumpfish swimming capacity [17]. Lumpfish welfare data were not collected for this study. However, the farmers at the site were aware of potential implications of currents, and had deployed shelters that were adapted for use in strong currents, providing firm surfaces to attach to as well as shelter from the current. Lines 417-423.

**319-323:** do you consider that handling by operators during bad weather conditions can also affect the welfare indicators monitored?

*In this study, fish were rarely handled at all other than the biweekly louse counts that were carried out on 10 fish per cage. In extreme conditions, these counts were postponed, but some were carried out in relatively large waves. However, the conditions on the farm were unlikely to have additional effects on the OWIs beyond what netting, immersion in anaesthetic bath, and manual handling would cause even in calm conditions.*

## 1.2 Reviewer 2

**L29:** Cage deformation, please explain.

*Changed to now read as follows:*

> Currents do not only affect the swimming behaviour and energy expenditure of salmon, they also affect the shape of salmon cages. Cage deformation due to current can cause a decrease in cage volume of 20% at 0.5 m $s^{-1}$ [6, 7]. While not as thoroughly studied, there are indications that waves too can change the shape of a salmon cage [8]. Now lines 38-41.

**L34:** ASC ?

*Aquaculture Stewardship Council, the aquaculture equivalent of MSC (Marine Stewardship Council) for wild caught fish. Full name and a reference now given in the MS:*

> This means that there is growing consumer pressure for not only environmental certifications such as ASC (Aquaculture Stewardship Council) [12], but also for assurances that the farms can deliver a minimum welfare standard, such as the RSPCA assured scheme [13]. Now lines 48-51.

**L32-36:** The authors are speaking about welfare here, and present some results about welfare state of fish. But the reader does not understand really the place of welfare in this study, since no statistics have been carried out, no link with other endpoints, and no discussion about welfare in discussion.

*The inclusion of fish welfare was only intended to be used as an indicator that conditions on the farm did not cause poor welfare as evidenced by 95% of OWI scores being 4 or less of the possible total of 10. However, the authors acknowledge that the OWIs as they stand are not well enough explained and the purpose for including them is not clear. We have clarified in text:*

> In order to ascertain the general welfare of salmon on the farm during the study period, we carried out Operational Welfare Indicator registrations. Now lines 165-166.

**L40:** "A combination of many environmental factors, [...] waves". Only waves and currents?

*We consider the separate wave and current parameters as different factors (wave height and period do not necessarily affect the fish in the same way), but this is perhaps not clear from the text. We have changed it and it now reads like this:*

> This study is an attempt at detailing how salmon are affected by the combined effects of waves and currents. Particularly, how the hydrodynamic conditions change their preferred positioning in the cage and how they change their behaviour. Now lines 55-58

M&M:
Overall, the M&M needs more details. No reference about material used is provided by the authors. Sensors, videos camera, etc. This has to be provided according to the journal guideline. Mostly, the M&M lacks major information about how data were acquired by the authors Please define swimming effort, shoal cohesion.

*We have tried to provide more information in the methods section and we have also clarified where to find the relevant information. We are unsure of what the reviewer means by reference about the material. Hopefully the clarified methods suffice.*

**L42:** Please detail more which parameter you monitored on fish.

*Done. Now reads:*

> Here, we monitor the behaviour of salmon and how they use the space available to them in a cage exposed to both currents and waves throughout the winter months of 2019/2020. Lines 59-61.

**L68:** please provide reference of material used.

*We have detailed the types of equipment used in Table 1. However, we did not make that clear in text, so now we reference the table when we mention equipment. There was information missing on our cameras, which has now been added. Is this sufficient information, or should we be providing more?*

**L70:** To monitor waves and currents what?

*Wave period and height and current speed and direction. Now added.*

> To monitor wave height and period and current speed and direction,... Now line 93.

**L72:** wave data, current data? It is not clear for the reader what you are measuring

*Hopefully, the line above makes this clearer*

**L77:** and figure 2: Echo sounders did not cover all the cages? I do not understand. Caption Figure 2: The caption needs more details to understand the symbol on the figures.

*The echo sounders did indeed not cover the entire horizontal area within the cage. They have quite narrow beams and are best suited to monitor a specific area within a salmon cage. This is also why we have two echo sounders monitoring two locations within the cage rather than one echo sounder covering the entire cage. We have clarified the legend.*

**L87:** Jumping is not analyzed by the authors? As well feeding? Why do mention it in M&M if not analyzed?

*Indeed. We have removed any mention of monitoring these behaviours.*

**L109:** It is not very clear how video recording and analysis is managed by the authors. They are recording baseline of 5 minutes once a week, and then 3-4 time a day? (Without writing how long these captures lasted). And later how it was managed for the analysis? Are all the videos analyzed? Seems not. This needs to be clearly explained by the authors. How duration analyzed? How it is compared paired with wave current data?

*We agree that this was not very clearly explained. We have made an attempt to clarify the video selection process. The reviewer correctly deduced that not*

*all videos recorded were used for analysis. This was mainly because a lot more than necessary video was recorded, which resulted in a lot of video representing very similar conditions. In order to avoid redundancy, we aimed to analyse a representative 30 videos per camera (which would result in 150 videos) in addition to the baseline videos, but at times, some video feeds were obscured due to the conditions at the farm (particularly the north camera). Therefore, some cameras were used more than others to compensate for this and the resulting 165 videos represent, so well as we are able, the full range of locations and hydrodynamic conditions within the cage.*

> Video cameras were remotely controlled using iSpy [18] and scheduled to record for five minutes each once per week. In addition to these baseline recordings, alternative schedules were enacted for bad weather events to record for five minutes three to four times each day during bad weather to capture behaviours in large wave conditions. At the end of the trial, the recorded wave data were sorted by wave height and period length and videos representing the full range were selected for analysis. Similarly, videos from days with weak current and strong current were also chosen for analysis, particularly from days where several videos were recorded within a day in order to capture the tidal current. This selection process resulted in 22 baseline videos in addition to 143 videos chosen to represent different hydrodynamic conditions. Unfortunately, when the weather was very bad, power did sometimes cut out, so it was not possible to assemble a perfectly balanced number of videos for all conditions. For swimming effort, most of the videos selected for behavioural observations were used, but in some videos, no fish were present, so these two data sets are not the same size. Now lines 144-157.

**L115-118:** Continuously, what's mean? More information is needed about data acquisition. Frequency of data acquisition? Mean on a period? how calculations are done? Please provide more information about it. This is important information to judge about the validity of the experiment.

*The authors mention in the section about equipment setup the frequency of pings (once every 4 seconds). Continuously here means that every ping is recorded onto the local hard drive immediately. Data were uploaded into the cloud on an ad-hoc basis and all further processing is carried out after the fact as detailed in "data processing"*

> To monitor vertical distribution of salmon within the cage, two echo sounders (Table **??**) were attached to the cage bottom, both positioned half way between the centre and side of the cage at opposite sides parallel to the coast line. The echo sounders were suspended from the bottom of the cage looking up, thereby recording distance to the surface as well as any fish within the echo

> sounder beam. This allowed us to measure the depth of the cage as well as which parts of the water column were occupied by fish. The echo sounders were set up to ping once every four seconds for the duration of the experiment. Lines 101-108.

**L121:** Please provide a reference to support this use of welfare indicators.
and
**L124:** Please provide concentration of MS-222 used for anesthesia.
and
**L123:** for each cage? not only one cage monitored?

*We did indeed collect OWIs from all the cages. As mentioned by the other reviewer, the number of fish for this monitoring is quite low, so we chose to monitor all of the cages, which were exposed to fairly similar conditions, rather than just one cage. The methods have been changed and now read as follows:*

> In order to ascertain the general welfare of salmon on the farm during the study period, we carried out Operational Welfare Indicator registrations. During the sampling period, Operational Welfare Indicators (OWIs) were recorded every two weeks when weather allowed (Table **??**). Prior to this, welfare indicators were collected on a more ad-hoc basis to establish a baseline, and finally again at harvest. The large gap in data from March until harvest is due to the COVID-19 pandemic preventing fieldwork. The OWIs were adapted by Hiddenfjord from SWIM 1 and 2 [14, 15] to make them more practical to use as part of regular farm management practices. The OWIs were collected from 10 salmon from each cage in connection with routine louse counting. The salmon were caught using a dip net, anaesthetised in 60 mg $L^{-1}$ Finquel (MS-222) and lice numbers and gill condition were recorded before OWIs were recorded. After regaining consciousness in fresh seawater, the salmon were released back into the cages. For the purposes of this study, a sum of scores for each salmon excluding sclera colour was used to determine the overall welfare of the salmon. Sclera colour or eye darkening was not included here because while there is evidence that they can be used as an indicator of stress [16], this has not yet been well established in salmon. The total score excluding sclera can range from 0 to 10 with a low number indicating good welfare and a high number indicating poor welfare. Many individual based indicators in SWIM 1 and 2 are not included because there was either no variation in them (e.g. sexual maturation did not occur) or they did not relate to conditions on the farm at the time (e.g. deformities). Lines 165-184.

**L130:** jumping behavior, feeding and presence? Jumping behavior and feeding analyzed? it seems not. Please delete if these variables are not then analyzed.

*This has been removed from the manuscript as it does not reappear in the results*

**L138:** How did the authors define the fish shoaling? Arbitrary? some parameters need to be implemented in BORIS software?

*This has hopefully been explained a bit more clearly now. There is no programming in Boris except writing up an ethogram of all the behaviours that you would like to record while watching the video.*

> Due to the nature of salmon moving in and out of camera, no attempt at counting the salmon was made. Instead, videos were coded in one of three qualitative categories throughout; "No salmon" (less than five salmon visible), "Some salmon" (more than five and less than 50 salmon visible), and "Many salmon" (More than 50 salmon visible). Finally, the presence of salmon near the surface was recorded for reasons of validating the near surface echo sounder data. When salmon were recorded in cameras with a view of the net, collisions with the net were also recorded.
>
> In addition to the presence of salmon, the general behaviour of the salmon was recorded. Again here, we did not use a focal animal or try to record the behaviour of every animal. Instead, when more than 80% of the salmon performed the same behaviour (such as shoaling, standing on current, or directional swimming), that is what was recorded for the duration. When there was no clear majority behaviour in frame, no behaviour was recorded. Typically, this meant that the salmon were swimming in no coherent shoal with some salmon standing on the current and some salmon swimming in one direction and some other swimming in another direction. All video segments not classified as "standing on current" were classified as "free swimming" and all video segments where salmon were not together in a coherent shoal were classified as "not shoaling".
>
> Finally, swimming effort was recorded by noting the time taken to beat with the tail fin three times by three different fish in each video. Now lines 186-205.

**L139:** please, could you explain a bit more how this analysis has been managed.

*This has been described in more detail now. Hopefully the above has clarified how the data were recorded from the videos.*

**L151:** swimming mode? not verry clear how the authors analyzed the data. Neither clear in the result section.

*The shift between swimming freely in the cage and "standing on current" now replacing "Keeping place against the current" has now been described in the introduction. Similarly, the methods are now more clear on how behaviours were recorded.*

**L156:** How other parameters of interest related to swimming (e.g. shoal cohesion, swimming effort) were analyzed by the authors?
"Nearness to camera was [...] intercept term?" I do not understand. For the modelling part, how the authors check the condition of use of the different models fitted?

*Swimming effort was analysed using a linear mixed model with tail beats per second as the dependent variable and current speed, direction and wave period as predictor variables. Interactions were found between current speed and direction as well as current speed and wave period, so these two interactions are included in the minimal model. Camera was used as random intercept term. Results on shoal cohesion now mentioned in the manuscript. For clarity the methods have been changed, and now read:*

> For video data, the following methods were used; to analyse swimming mode, a general linearised mixed effects model was used with a binomial (log link) family where each video was classified as salmon being mostly in either swimming mode, with current strength and current direction as predictors, and with camera as the random intercept term. The effects of environmental conditions on swimming effort was analysed using a linear mixed effects model with tail beats per second as the dependent variable, current speed, current direction, and wave period as predictors as well as camera as random intercept term. The reason for including camera as random intercept term in these models is that hydrodynamic conditions are not uniform throughout the cage, so the salmon in the different cameras will be affected differently by the conditions measured outside of the cage. The models described are the minimal adequate models, where variables that did not significantly affect the fit of the model have been removed. The amount of time where "Many" salmon appear in a camera (proxy for proximity to sides and surface) was analysed using a general linearised mixed effects model with time where "Many" salmon were visible in the camera as dependent variable, wave height (Hm0) and period (Tp) as predictors, and cameras as random intercept term. To assess the effect of hydrodynamic conditions on salmon collisions with the cage net, a poisson family general linearised model was used. The dependent variable was the number of collisions divided by the amount of time where there were salmon visible, to get a measure of collisions per second, multiplied by 300 to extrapolate to a 300 second (five minutes) long

observation, and then rounded to whole number in order to use a poisson family model. Predictors were current speed and wave height. Shoal cohesion was investigated using a binary classification of videos to "shoaling" and "not shoaling" in a binomial family glm due to the highly bimodal nature of this variable where salmon were either mostly shoaling or not at all. Predictors were wave height, current direction and camera. Model fit for each model was assessed using diagnostic plots. Lines 222-247.

**Results:**

**Figure 5:** please show all points in the figure you used to plot the linear regression. Please provide the results of the statistics in the results section.

*The data extracted from the pressure sensors is only included to illustrate that there is cage deformation. All of the points have been plotted, though with quite a high alpha in order to show more of the data that would otherwise be obscured by overlying data points. We have not done a linear regression on these data, as they seem pretty clear to us and the focus of this manuscript is not to investigate cage deformation. This has already been done and we simply included pressure sensors in our fieldwork to document the extent of deformation at this site and to verify the depth measured by the echo sounders.*

**L208:** Please, provide the standard errors of the models, and the pseudo R2 if possible. It looks like that the model for the Northbound does not fit very well the data. About cage deformation, the statement of the authors is provided without any statistic. In addition, the figures provided by the authors are interesting but without statistics and raw data (points in the figures that supports the different regression). Without this, the reader cannot judge about the validity of the author's statement.

*The theoretical R squared is 0.279 (Marginal) and 0.525 (Conditional). What is not shown in the plot is that the model is mixed with random intercepts per camera. For simplicity, all of the data from all cameras have been plotted in one figure, which is probably why the fit looks worse. The estimates for the two variables are for current speed: 17.9 (SE 4.9) and for direction (southbound): -2.4 (SE 0.6). We have clarified how the model was built in the methods (mentioned above) and also clarified the figure legend to reflect this. In terms of other figures, the only ones not presenting regression fits or other descriptive statistics are the raw echo sounder figures. We have chosen to present the data in this way because it illustrates much more clearly how the fish are responding to the variables than would regression fits such as those presented in [9].*

The caption of figure 5 is not adapted. It is material and methods. The caption does not describe the figure.

*Noted. We have changed it, and it now reads:*

> Depth measured by pressure sensors located at the bottom of the side net (Side- west, south, east, and north) and half way from the side to the centre (Bottom- west, south, east, and north). Points are depths measured and lines are lines of best fit. Darker points and lines are depths in southbound current and light points and lines are depths in northbound current.

Figure 6 captions: please provide more information about model predictions drawn in the caption. "points are proportion of time where the majority of fish were keeping place against the current", why not analyzing the proportion of fish ? It seems more sound, isn't it ? We do not really know how videos were analyzed ? all videos ? how much minutes by videos? Always same time for a video ?

*If we used images rather than video, the proportion of fish in each image could be analysed. However, because we used video, getting the exact proportion at any one time was impossible, so videos were coded as "standing on current" when an estimated 80% or more of the fish in frame were performing this behaviour. The proportion of time in the video that this was the case was then used for analysis. Hopefully, the methods now make it clearer how the videos were analysed. The lines have been more carefully described:*

> Points are proportion of time where the fish were standing on current in a video. Lines represent model predictions of the probability of a video having fish standing on current a majority of the time. Model predictors are current speed and direction. Lighter colour signifies a northbound current direction whereas a darker colour signifies a southbound current direction.

**L226:** Some illustration of the authors statement?

*for the sake of brevity, many figures were not included in this manuscript. However, we are happy to include one on horizontal preference within the cage. As a side note, we noticed that our statement about the "East" camera was erroneous as the increase was smaller from 50% to 76% and not to 100%.*

**L256-260:** Do the authors perform some statistics on the welfare score ? Does the welfare score change from date to authors ? This could be linked to wave, currents ? If not, it is difficult to understand what are the aim of showing the welfare results.

*Welfare scores changed significantly over time with the three harshest winter months scoring significantly higher than the rest of the observations. We have added this statistic to the results and discuss this effect further in the discussion.*

**Discussion:**
The discussion "discusses" the results of the study without providing any references from other work (only a previous study from the same authors), which is insufficient for a research paper. The results of the study need to be put in relation with more studies. In addition, some of the results are not discussed at all (e.g. welfare score).

*The authors acknowledge that the discussion needed some work. We have discussed the relevant literature now to provide some perspective and compare our results with other studies.*

**L264-266:** This needs to be reformulated.

*We have substantially rewritten the discussion:*
Figures and captions: The captions need to clearly define what is on the figures. In the majority of case, the captions are giving unnecessary details, while lacking necessary details to understand the figure. This needs to be checked and corrected in the whole manuscript. Figures in the correct order would be appreciable for reviewer's work.

*The figure legends have been thoroughly reviewed in order to ensure that they contain all relevant information and no irrelevant information. We hope they are clearer now.*

# References


1. Wright D, Glaropoulos A, Solstorm D, Stien L, Oppedal F. Atlantic salmon Salmo salar instantaneously follow vertical light movements in sea cages. Aquaculture Environment Interactions. 2015;7(1):61–65. doi:10.3354/aei00136.

2. Oppedal F, Juell JE, Tarranger GL, Hansen T. Artificial light and season affects vertical distribution and swimming behaviour of post-smolt Atlantic salmon in sea cages. Journal of Fish Biology. 2001;58(6):1570–1584. doi:10.1111/j.1095-8649.2001.tb02313.x.

3. Oppedal F, Juell JE, Johansson D. Thermo- and photoregulatory swimming behaviour of caged Atlantic salmon: Implications for photoperiod management and fish welfare. Aquaculture. 2007;265(1-4):70–81. doi:10.1016/j.aquaculture.2007.01.050.

4. Solstorm F, Solstorm D, Oppedal F, Olsen R, Stien L, Fernö A. Not too slow, not too fast: water currents affect group structure, aggression and welfare in post-smolt Atlantic salmon Salmo salar. Aquaculture Environment Interactions. 2016;8:339–347. doi:10.3354/aei00178.

5. Johansson D, Laursen F, Fernö A, Fosseidengen JE, Klebert P, Stien LH, et al. The Interaction between Water Currents and Salmon Swimming Behaviour in Sea Cages. PLoS ONE. 2014;9(5):e97635. doi:10.1371/journal.pone.0097635.

6. Klebert P, Patursson Ø, Endresen PC, Rundtop P, Birkevold J, Rasmussen HW. Three-dimensional deformation of a large circular flexible sea cage in high currents: Field experiment and modeling. Ocean Engineering. 2015;104:511–520. doi:10.1016/j.oceaneng.2015.04.045.

7. Lader P, Dempster T, Fredheim A, Jensen Ø. Current induced net deformations in full-scale sea-cages for Atlantic salmon (Salmo salar). Aquacultural Engineering. 2008;38(1):52–65. doi:10.1016/j.aquaeng.2007.11.001.

8. Kristiansen D, Lader P, Jensen Ø, Fredriksson D. Experimental study of an aquaculture net cage in waves and current. China Ocean Engineering. 2015;29(3):325–340. doi:10.1007/s13344-015-0023-1.

9. Johannesen Á, Patursson Ø, Kristmundsson J, Dam SP, Klebert P. How caged salmon respond to waves depends on time of day and currents. PeerJ. 2020;8:e9313. doi:10.7717/peerj.9313.

10. Dam SP. A study on Atlantic Salmon (Salmo salar) in aquaculture: moving into exposed waters, the effect of waves on behaviour and growth.; 2015.

11. Oppedal F, Vågseth T, Dempster T, Juell JE, Johansson D. Fluctuating sea-cage environments modify the effects of stocking densities on production and welfare parameters of Atlantic salmon (Salmo salar L.). Aquaculture. 2011;315(3-4):361–368. doi:10.1016/j.aquaculture.2011.02.037.

12. ASC. Aquaculture Stewardship Council; 2021. Available from: https://www.asc-aqua.org/.

13. Assured R. RSPCA welfare standards for farmed Atlantic salmon; 2021. Available from: https://business.rspcaassured.org.uk/rspca-welfare-standards/.

14. Stien LH, Bracke MBM, Folkedal O, Nilsson J, Oppedal F, Torgersen T, et al. Salmon Welfare Index Model (SWIM 1.0): a semantic model for overall welfare assessment of caged Atlantic salmon: review of the selected welfare indicators and model presentation: Salmon Welfare Index Model (SWIM 1.0). Reviews in Aquaculture. 2013;5(1):33–57. doi:10.1111/j.1753-5131.2012.01083.x.

15. Pettersen JM, Bracke MBM, Midtlyng PJ, Folkedal O, Stien LH, Steffenak H, et al. Salmon welfare index model 2.0: an extended model for overall welfare assessment of caged Atlantic salmon, based on a review of selected welfare indicators and intended for fish health professionals. Reviews in Aquaculture. 2014;6(3):162–179. doi:10.1111/raq.12039.

16. Freitas RHA, Negrão CA, Felício AKC, Volpato GL. Eye darkening as a reliable, easy and inexpensive indicator of stress in fish. Zoology. 2014;117(3):179–184. doi:10.1016/j.zool.2013.09.005.

17. Hvas M, Folkedal O, Imsland A, Oppedal F. Metabolic rates, swimming capabilities, thermal niche and stress response of the lumpfish, *Cyclopterus lumpus*. Biology Open. 2018;7(9):bio036079. doi:10.1242/bio.036079.

18. ltd D. iSpy; 2019. Available from: ispyconnect.com.
