# Supplementary material for: Waves and currents decrease the available space in a salmon cage
Source: PLoS One. 2022 Feb 25;17(2):e0263850. doi: 10.1371/journal.pone.0263850 (PMC8880890; doi:10.1371/journal.pone.0263850)
Supplement: Attachment — Submitted filename: Response to reviewers.pdf [file pone.0263850.s002.pdf]

1 Response to reviewers

The authors thank the reviewers for considering our manuscript again and are very pleased that the changes made were considered sufficient.

1.1 Reviewer 1

In the previous version of the manuscript, I had some major concerns, mainly about missing details concerning methods and analysis, as well an insufficient discussion of the results. In the revised version, Ása Johannesen et al. did a careful revision of the manuscript, answering all of my concerns and those of the second reviewer. I want to congratulate them for their work, and so my recommendation is to accept the manuscript.

Thank you! We really appreciate your careful review.

Minor comment:
Ln 415 : please, could you provide a reference to support the statement that such current spped might actually perform the role of environmental enrichment.

We have added two references to support our statement, though the authors acknowledge that literature on this subject is rather sparse. The references that have been added are.

Williams, T.D., Readman, G.D., Owen, S.F., 2009. Key issues concerning environmental enrichment for laboratory-held fish species. Lab Anim 43, 107–120. https://doi.org/10.1258/la.2007.007023

and

Arechavala-Lopez, P., Cabrera-Álvarez, M.J., Maia, C.M., Saraiva, J.L., 2021. Environmental enrichment in fish aquaculture: A review of fundamental and practical aspects. Rev Aquac raq.12620. https://doi.org/10.1111/raq.12620

1.2 Reviewer 2

The authors have followed the suggestions in a very satisfactory way, and I therefore recommend this work for publication.

Thank you!
